# Missing Girls in India: Infanticide, Feticide and Made-to-Order Pregnancies? Insights from Hospital-Based Sex-Ratio-at-Birth over the Last Century

**DOI:** 10.1371/journal.pone.0002224

**Published:** 2008-05-21

**Authors:** Mohit Sahni, Neeraj Verma, D. Narula, Raji Mathew Varghese, V. Sreenivas, Jacob M. Puliyel

**Affiliations:** 1 Department of Neonatology and Pediatrics, St. Stephens Hospital, Delhi, India; 2 Department of Biostatistics, All India Institute of Medical Science, New Delhi, India; JARING, Malaysia

## Abstract

**Background:**

There are 44 million missing women in India. Gender bias; neglect of girls, infanticides and feticides are responsible. The sex ratio at birth can be used to examine the influence of antenatal sex selection on the sex ratio.

**Materials and Methods:**

Records from 321,991 deliveries at one hospital over 11 decades were utilized. The middle year in each decade was taken as representative of the decade. Data from 33,524 deliveries were then analyzed. Data for each decade was combined with that of previous decades and compared to the data of subsequent decades to look for any change in the trend. Sex ratio in the second children against sex of the first child was studied separately.

**Results:**

The mean sex ratio for the 110 years examined was 910 girls to 1000 boys (95% CI; 891 to 930). The sex ratio dropped significantly from 935 (CI: 905 to 967) before 1979, to 892 (CI: 868 to 918) after 1980 (P = 0.04). The sex ratio in the second child was significantly lower if the first child was a girl [716 (CI: 672 to 762] (P<0.001). On the other hand, there was an excess of girls born to mothers whose first child was boy [1140 girls per 1000 boys (CI: 1072 to 1212 P<0.001)].

**Conclusions:**

The sex ratio fell significantly after 1980 when ultra sound machines for antenatal sex determination became available. The sex ratio in second children if the first was a girl was even lower. Sex selective abortions after antenatal sex determination are thus implicated. However data on second children especially the excess of girls born to mothers who have a previous boy seen in the decade before the advent of antenatal ultra sound machines, suggests that other means of sex selection are also used.

## Introduction

Data from the census of 2001 suggests that there are only 933 women for every1000 men in India [1]. In 1992 Amartya Sen calculated that 37 million women were ‘missing’ in India [2]. The UN in 2001 estimated that there were 44 million missing women in India [3]. Societal bias favoring males is responsible for the situation [4]. This bias manifests as neglect of girls and women resulting in their early death [5], [6], [7], female infanticide [8], [9] and more recently, antenatal sex determination and female feticide [10]. In India, ultra sound machines that permit non-invasive antenatal sex determination first became available in select centers in the late 1970s and became more widespread in the 1980s [11], [12]. Soon thereafter abortion of female fetuses were reported from many major cities of India [13], [14]. Several reports suggest that sex selective abortion became more common in the 1990s [15], [16]. Amartya Sen believes that the pattern of gender inequality shifted from ‘mortality inequality’ to what he calls ‘natality inequality’ due to female feticide after the facility for antenatal sex determination became available [11], [17]. Others suggest that parents are not substituting prenatal for post-natal discrimination against girls but combining the two strategies [18]. The relative contribution of these modes of discrimination, to the unbalanced sex ratio in India, is still unresolved [19].

The age specific sex ratio is used to determine the age at which women go ‘missing’. However infanticides, in the first few days, are often reported as still-births [19] or not reported at all within the incomplete birth registration system [9]. It is therefore difficult to assess the number of girl children ‘missing’ from the census data on account of sex selective abortion and the number lost due to early infanticide. Data on sex ratio at birth in hospital records can be crucial to estimate the influence of female feticide on the sex ratio as this is not affected by other factors like infanticide and neglect of girl children. This study was done to look at secular trends in the sex ratio at birth of babies born in a hospital over the last 110 years. We hypothesized that if female feticide is a factor affecting the sex ratio, there would be a fall in the newborn female to male sex ratio after the technology for antenatal sex determination became widespread in the1980s.

## Materials and Methods

Records of 321991 babies delivered at the hospital (St Stephens Hospital, Tis Hazari, Delhi India) from 1900 to 2006 were available with the hospital records office. Data pertaining to an additional, approximately 700 deliveries, for the period 1907 to 1910 could not be accessed. We used data from the middle year of each decade to represent the sex ratio during that decade and as such we studied records from 1 July 1904 to 30 June 1905 for the first decade in the century and so on for each of the subsequent 10 decades. Some pages in the records for 1914 and 1934 were damaged due to improper storing and so, for those two decades, data from 1915–16 and 1935–36 respectively were collected. Data from 33,524 deliveries was taken as representative of the 321,991 deliveries at the hospital and these were the subjects of the present study. Of these, 197 deliveries were excluded due to incomplete recordings, or because ambiguous genitalia made gender assignment uncertain. Still births and abortions were excluded. For multiple pregnancies, each live born baby was recorded as a separate delivery. Mother's age, religion, maternal education and details of previous pregnancies were noted. Sex ratio was studied for each decade as number of girl babies born for every 1000 boys. We looked for statistical significance comparing the sex ratio for each decade with the overall average sex ratio.

Data for each decade was combined with that of previous decades and compared to the data of subsequent decades to look for any change in the trend. For example in the analysis for changes after the fourth decade the century (1940–1949) we compared the sex ratio of babies born 1900–1949 with the sex ratio in babies born after that (1950–2005).

Data on previous pregnancies and sex of previous children were available in the records only after 1970. Sex ratio in the second children born to mothers was also studied. This sex ratio was examined in mothers whose first child was a boy and separately in those whose first child was a girl. Data on the education status of mothers was available only from 1985. Approval of the hospital research committee was obtained for this retrospective study of hospital records and recording of the data in an anonymized manner with serial numbers. Formal ethics approval was therefore not obtained.

Confidence interval (CI) for the sex ratios at different reference time periods were calculated and Chi-square test was used for assessing the statistical significance. Analyses of these were done using Stata version 9.1. To look for the difference in proportions of the sex ratios and their CI we used the software ‘Statistics with Confidence’ (www.som.soton.ac.uk).

## Results

**Figure 1:**
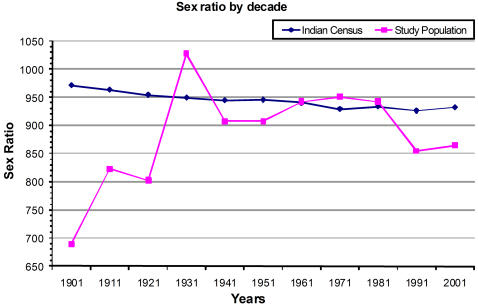
Sex Ratio by Decade.

Figure 1 shows the data from the census of India on sex ratio declining gradually over the century. The hospital based sex ratio in each decade is depicted alongside. Data for the period prior to 1930 comes from a relatively small sample so detailed analysis is done only for the period after 1930. Table 1 shows the sex ratio at birth in each decade and also the analyses comparing sex ratio in the period after the reference decade with the sex ratio till that point. Overall, there were 910 girls to 1000 boys (95% CI; 891 to 930). The sex ratio in 1995 was 855 (CI: 816 to 895) and this was significantly lower than the overall average sex ratio of 910. The sex ratio was 782 (CI: 675 to 905) up to 1929 compared to 913 (CI: 893 to 933) (P = 0.04) in the decades that followed. The next big change in the evaluation of trends (columns d e f) was seen in 1979. The sex ratio for the decades up to 1979 was 935 (CI: 905 to 967) and it was 892 (868 to 918) for the decades after 1980 (P = 0.04).

**Table 1:** Sex ratio at birth in every decade from 1905 to 2005.

Decade	Total number of deliveries	Sex ratio (95% C.I.)	P†	Sex ratio (95% C.I) Up to the Reference year	Sex ratio (95% C.I) Subsequent Years	P‡	Difference between sex ratios# (95% C.I.)
	(a)	(b)	(c)	(d)	(e)	(f)	
1900–1909	157	689		689	911	0.08	0.07
		(495 940)	0.08	(495 940)	(892 931)		(−0.01 to 0.11)
1910–1919	195	823	0.48	760	912	0.09	0.05
		(617 1089)		(613 937)	(892 931)		(−0.02 to 0.11)
1920–1929	375	803	0.23	782	913	0.04*	0.04
		(653 983)		(675 905)	(893 933)		(−0.01 to 0.09)
1930–1939	806	1028	0.08	904	910	0.89	0.00
		(895 1181)		(817 999)	(890 930)		(−0.03 to 0.04)
1940–1949	973	908	0.98	906	910	0.91	0.00
		(801 1030)		(838 980)	(890 931)		(−0.03 to 0.03)
1950–1959	1843	908	0.97	907	910	0.91	0.00
		(829 995)		(855 963)	(890 932)		(−0.02 to 0.02)
1960–1969	3629	943	0.31	923	906	0.46	0.00
		(884 1007)		(884 965)	(884 928)		(−0.02 to 0.01)
1970–1979	6033	951	0.12	935	892	0.04*	−0.01
		(904 1000)		(905 967)	(868 918)		(−0.03 to 0.00)*
1980–1989	8026	943	0.15	938	859	<0.01*	−0.02
		(903 985)		(913 963)	(828 891)		(−0.04 to–0.01)*
1990–1999	7231	855	0.02*	917	865	0.07	−0.01
		(816 895)		(896 938)	(814 918)		(−0.03 to 0.00)*
2000–2005	4256	865	0.12				
		(814 918)					
All years	33524	910 (891 930)					

† P value compared to the overall sex ratio (910) during 1905–2005.

‡ P value comparing column d & e.

# Difference in proportion of sex ratios in column d & e.

* Significant.

Table 2 looks at sex ratio in accordance with religion and Table 3 looks at sex ratio according to maternal education. There was no significant difference between groups.

Table 4 looks at sex ratios in second children depending on the sex of the first child. The sex ratio in the second child if the first was a girl fell to 716 (CI: 672 to 762) girls per 1000 boys. The difference from the overall sex ratio of 910 girls was statistically significant (P<0.001). In contrast to this, there was an excess of girls, if the previous child were a boy. The sex ratio was 1140 girls per 1000 boys (CI: 1072 to 1212) (P<0.001).

**Table 2:** Sex ratio according to the religion (1905–2005).

Religion	Total	Sex ratio (95% C.I.)	P‡
Hindu	30818	913 (893 933)	0.85
Muslim	1287	899 (805 1002)	0.83
Christian	455	865 (718 1039)	0.59
Sikh	801	833 (724 957)	0.22
Other	50	1174 (672 2107)	0.37
Not known	113	949 (652 1375)	0.83
All religions	33524	910 (891 930)	

P‡ Compared to the overall sex ratio (910) during 1905–2005.

**Table 3:** Sex ratio according to the mother's education (1985–2005).

Education	Total	Sex ratio (95% C.I.)	P‡
0–5 years of school education	2399	924 (853 1001)	0.71
6–10 years of school education	4973	913 (864 965)	0.91
11–12 years of school education	3821	857 (804 913)	0.08
College & above	8164	890 (852 930)	0.38
Education status not recorded	156	753 (543 1029)	0.24
Total	19513	892 (868 918)	0.28

P‡ Compared to the overall sex ratio (910) during 1905–2005.

**Table 4:** Sex ratio among the second order babies, depending on gender of first born.

A	B	C	D
Year	Sex ratio (95% C.I.) when first child is female.	Sex ratio (95% C.I.) when first child is male.	Difference between sex ratios#.
	[N = Total number]	[N = Total number]	(95% C.I.)
1974–1975	754 (658 862)	1266 (1107 1452)	0.13
	P<0.01*	P<0.001*	(0.08 to 0.18)*
	[N = 854]	[N = 852]	
1984–1985	865 (773 967)	958 (858 1070)	0.03
	P = 0.38	P = 0.37	(−0.01 to 0.06)
	[N = 1225]	[N = 1255]	
1994–1995	610 (541 686)	1213 (1085 1357)	0.17
	P<0.001*	P<0.001*	(0.13 to 0.21)*
	[N = 1167]	[N = 1239]	
2004–2005	629 (541 728)	1222 (1059 1412)	0.16
	P<0.001*	P<0.001*	(0.11 to 0.21)*
	[N = 741]	[N = 753]	
All years	716 (672 762)	1140 (1072 1212)	0.12
	P<0.001*	P<0.001*	(0.09 to 0.14)*
	[N = 3987]	[N = 4099]	

P value compared to the overall sex ratio of 910 for the period 1905–2005.

# Difference in proportion of sex ratios in column B & C.

* Significant.

## Discussion

Our study shows that the sex ratio at birth fell significantly in 1995 to 855 (girls per 1000 boys) when comparisons were made to the overall average sex ratio of 910 seen over the eleven decades. While looking for trends we found that the sex ratio for the period ‘1979 and earlier’ was significantly higher [935 (CI 905 to 967)] than the period ‘1980 and after’ [892 (CI 868 to 918) P = 0.04]. This fall in sex ratio coincided with the availability of ultra sound for antenatal sex determination [4], [12].

The Pre-natal Diagnostic Techniques (Regulation and Prevention of Misuse) (PNDT) Act that made antenatal sex determination and sex selective abortion illegal, was passed in 1994. It came into effect in 1996 [20]. Our data shows the sex ratio in 2005 (865 CI: 814 to 918) was not very different from that in the decade before the PNDT act came into effect (855 CI: 816 to 895). This suggests that the Act has had little impact on the problem. This is similar to the findings of others [21], [22], [23].

The sex ratio for the period prior to 1929 was 782 (CI: 675 to 905) compared to 913 (CI: 893 to 933) (P = 0.04) in the decades that followed. However the sample size for the period prior to 1929 was small and the CI for the difference in proportion between sex ratios was not significant at the 95% level of confidence.

Our data showed no relation between the sex ratio and religion or years of education of the mother. This is in agreement with the findings of others who have shown that gender bias exists regardless of religion, caste and socio-economic class, although it seems that it is more prevalent among the middle classes compared to the poor [3], [7]. In our study we made no comparison between income groups.

The concept of ‘missing women’ was highlighted by the Nobel laureate Amartya Sen to focus scholarly and public attention on this social problem [2]. He had said at that time that there were 100 million missing women world-wide of whom 37 million were missing in India. Using more sophisticated methodology, Klasen estimated in 1994 that the correct figure was 90 million missing women worldwide and that it would be 100 million by the year 2000 [24]. Ordinarily females have greater resistance to disease and overall greater longevity, so in circumstances where they have the same nutrition and healthcare (as males), females have lower mortality across all age groups [2]. The bleak situation for men is compounded by a greater tendency to engage in risk behaviour and violence which increases their risk of premature mortality [25]. This leads to there being more women than men. Sen in his original computation had used the female: male ratio of 1.022 observed in sub-Saharan Africa as the standard for comparison [2]. A decade after he made his original observation on missing women, Sen wrote in 2003 that female disadvantage in mortality had reduced substantially but this was counterbalanced by a new female disadvantage–that of natality through sex specific abortions against female fetus [11]. Compared to normal ratio of about 950 girls being born per 1000 boys as observed in Europe and North America, the ratio was 920 in Singapore and Taiwan, 880 in Korea and 860 in China. Data in this study suggests that the sex ratio in India after 1995 was similar to that in China (approximately 860) [11].

Biological explanations for some of the missing women have been offered. Nobel Laureate BS Blumberg has proposed that Hepatitis B can influence sex ratio. Carriers of hepatitis B have offspring sex ratio around 1.5 boys for each girl[26], [27], [28], [29]. Emily Oster [30] used this information alongside the Hepatitis B carrier rate in India to suggest that Hepatitis B can explain 20% of the missing women in India. The model however ignores the suggestion by Blumberg that while hepatitis B infection leads to an excess of boys, immunity to Hepatitis B moves the sex ratio in the opposite direction such that persons with anti-HBsAg tend to have more girls. The numbers of people with naturally acquired anti-HbsAg are much higher than the HbsAg carriers in India. In one study HbsAg carrier rate ranged from 0.9 to 4.1% but anti-HbsAg ranged from 12.9% to 18.4% [31]. This high prevalence of anti-HbsAg should have caused an excess of girls. Hepatitis B is therefore an unlikely explanation for the missing girls.

Sex ratio at birth is also influenced by a host of other factors including infections like toxoplasma infection [32], smoking [33], maternal nutrition [34], and hormonal factors during pregnancy [35], [36]. There may be differences in the prevalence of these factors in different populations and this may be responsible for the differentials in sex ratio seen world wide.

Jha et al found the adjusted sex ratio in India, if the first child was a girl was 759 per 1000 males and in contrast to this, it was 1102 females per 1000 males if the first were a boy [37]. This was evidence of sex selective feticide but could also result from non-reporting of infanticides or misclassification of infanticides as still births. Our study done on hospital deliveries was expected to obviate the misclassification problem. The study by Jha and the correspondence that followed showed up an interesting paradox. The research looking for ‘missing girls’, discovered also ‘missing boys’ (among families who have already had a boy). George in his comments on the paper by Jha et al has written that it would appear from the paper that 200,000 male feticides take place in India annually among those who have had a previous boy child [38]. As a researcher and activist, George writes that he has traveled to many parts of India looking for sex-selection practices but he has not come across selective male feticide reported even anecdotally. His conclusion was that the only plausible reason for Jha and colleagues' unusual birth order distribution was systematic undercounting of live-born girls.

Our study also showed the same trend as shown by Jha et al.–that more girls are born as second children, if the first child was a boy [1140 girls (CI: 1072 to 1212) if the first child was a boy compared to 716 girls (CI: 672 762) born as second children when the first child was a girl. This trend was seen even in the data prior to 1980 (during an era when facility for antenatal sex determination was not widely available) [12]. This excess of girls born to families with previous boys is unlike data from other countries like China where the sex ratio in second children ‘normalized’ after the birth of the first boy. In China, Yi et al found that women whose first child was a daughter had a much greater chance of having a boy the next time (sex ratio 670 girls to 1000 boys) but the chance of having a girl if the first child was a boy was not significantly higher than the natural sex ratio at birth (sex ratio 987 girls to 1000 boys) [39]. The phenomenon wherein there is an excessive number of girls (even higher than the natural sex ratio at birth considered as 950–1000 girls to 1000 boys) born as the second children to families with a previous boy thus seems peculiar to India and dates back to a period when antenatal sex determination was not feasible. Further qualitative research is needed to elucidate the methods used in this form of sex selection.

We have looked at sex ratio at birth over 110 years. We used hospital delivery data and so we can be confident the figures are not corrupted by infanticides. Our study however has one notable weakness. It relates to sex ratio of children born in a hospital. According to the National Family Health Survey 2 (1998–1999), two thirds of deliveries in India take place at home and outside of medical institutions. Our data cannot therefore be said to be representative of India. Further it may also be argued that if the sex of the child is known antenatally, there is a greater chance that male fetuses will be brought to the hospital for delivery and this could alter the ratio. This may be seen as an antenatal extension of the practice wherein boys are presented earlier in their illness and more frequently to the hospital [6]. However the data from second children delivered at this hospital shows that the majority of children are girls if the previous child was a boy, and this militates against the suggestion that boy fetuses are selectively bought to hospital for delivery.

In conclusion, our data shows there was a fall in the sex-ratio at birth, temporally coinciding with the widespread availability of the technology for antenatal sex determination. The PNDT act has not improved the situation. There is therefore reason to further invigorate the campaign against this unethical practice.

Furthermore and more notably our study of second children born before 1980 suggests that sex selection practices must have been in vogue prior to the advent of antenatal sex screening. This form of sex selection has mostly preferred boy children but has also manifested itself as a preference for girl children in some families with a previous boy. It is reasonable to assume that these traditional methods of sex selection have not been abandoned in the face of the availability of the more definitive technique of antenatal sex determination and selective abortion. More research is needed to understand these traditional methods.

It is evident that mere legislation and the prosecution of a few high profile cases under the PNDT act cannot solve this social evil. Moves to address all forms of gender inequality with equal social and economic rights for males and females including the rights of inheritance are needed to strike at the causes for distortion of the sex ratio [40].
